# Structure and density of basaltic melts at mantle conditions from first-principles simulations

**DOI:** 10.1038/ncomms9578

**Published:** 2015-10-09

**Authors:** Suraj Bajgain, Dipta B. Ghosh, Bijaya B. Karki

**Affiliations:** 1 Department of Geology and Geophysics, Louisiana State University, Baton Rouge, Louisiana 70803, USA; 2 School of Electrical Engineering and Computer Science, Louisiana State University, Baton Rouge, Louisiana 70803, USA; 3 Center for Computation and Technology, Louisiana State University, Baton Rouge, Louisiana 70803, USA

## Abstract

The origin and stability of deep-mantle melts, and the magmatic processes at different times of Earth's history are controlled by the physical properties of constituent silicate liquids. Here we report density functional theory-based simulations of model basalt, hydrous model basalt and near-MORB to assess the effects of iron and water on the melt structure and density, respectively. Our results suggest that as pressure increases, all types of coordination between major cations and anions strongly increase, and the water speciation changes from isolated species to extended forms. These structural changes are responsible for rapid initial melt densification on compression thereby making these basaltic melts possibly buoyantly stable at one or more depths. Our finding that the melt-water system is ideal (nearly zero volume of mixing) and miscible (negative enthalpy of mixing) over most of the mantle conditions strengthens the idea of potential water enrichment of deep-mantle melts and early magma ocean.

## Abstract (web summary)

Our understanding of the origin and stability of deep-mantle melts depends on our knowledge of the component silicate liquids. Here, the authors model the effects of iron and water on melt structure and density, with results that support potential water enrichment of deep-mantle melts and an early magma ocean.

## Graphical abstract

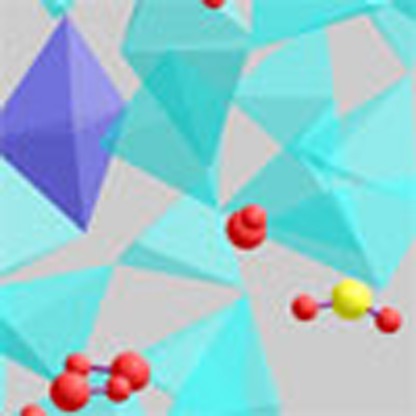

Among key melt properties of relevance for the chemical and thermal evolution of the Earth^1^,^2^,^3^ are the structure and density of molten silicates, which still remain unknown or poorly constrained over most of the mantle pressure and temperature conditions. Density contrasts between silicate liquids and solid mantle essentially control the stability and mobility of melt at depth^4^,^5^,^6^. Structural changes due to pressure can dramatically influence the melt density, and other properties including the melt viscosity and incompatible element partitioning. Extant experimental measurements provide limited information on these issues^7^,^8^,^9^. For instance, recent *in-situ* X-ray diffraction^9^ at pressures up to 60 GPa has characterized the structural changes in molten basalt only in terms of Si–O coordination with some assumption made about Al–O coordination, and perhaps with limited resolution due to broad, overlapping diffraction peaks. No information about other coordination environments could have been extracted. It is also not clear how the pressure-induced changes in the melt density are sensitive to composition at high pressure. In particular, iron and water are among the most important components^9^,^10^,^11^. Therefore, reliable quantitative estimates of their effects on the structure and density of silicate liquids as a function of pressure are essential.

Complementary to difficult experimentation at the conditions of deep interior is first-principles computation^12^,^13^,^14^, which has taken on an increased significance in the study of the silicate liquids, yet limited to compositionally simple systems. However, natural melts represent a multi-component CaO–MgO–FeO–Fe_2_O_3_–Na_2_O–K_2_O–Al_2_O_3_–TiO_2_–SiO_2_ system with volatile components including water^15^,^16^. Here, we study three basaltic systems in the temperature range of 1,800–4,000 K at typical mantle pressures by performing computationally intensive first-principles molecular dynamics simulations (see Methods section later). They include pure and hydrous phases of model basalt (MB) – the eutectic composition of 36 wt% anorthite and 64 wt% diopside, which differs from actual basalt in that it contains excess Ca to compensate Fe. MB is widely considered as a good analogue for natural basalt^7^,^8^,^17^. Our third melt composition is near-MORB (mid-ocean-ridge basalt) containing 9.9 wt% FeO, similar to experimentally studied molten basalts^9^,^11^. These simulations allow us to investigate the role of Fe and H_2_O in magmatic processes through accurate prediction of relevant bulk properties and access to microscopic (atomistic) information^12^,^13^,^14^,^17^.

Key questions that need to be answered in a quantitative manner are: how are various cations and anion coordinated at different pressures? Can coordination changes be linked with melt densification? Is water actually soluble in high-pressure melt? Can the melt density exceed the mantle density? Our simulations of three basaltic melts here suggest that all types of coordination between major cations (Al, Ca, Fe, Mg, Na and Si) and anion (O) increase strongly on compression with most changes occurring at the pressures below 30 GPa. They also reveal that the speciation of H_2_O component consists of mostly hydroxyls and molecular water at low pressure, which change to interpolyhedral (–O–H–O–) linkages and other extended forms at high pressure. The effects of the Fe and H_2_O components on density are such that the melts including hydrous melt may be buoyantly stable at one or more depths. Our work also represents direct (first principles) evidence for the possibility that the water component shows ideal mixing of volume as well as high solubility in high-pressure silicate melts.

## Results

### Atomic coordination in silicate melts

**Figure 1:**
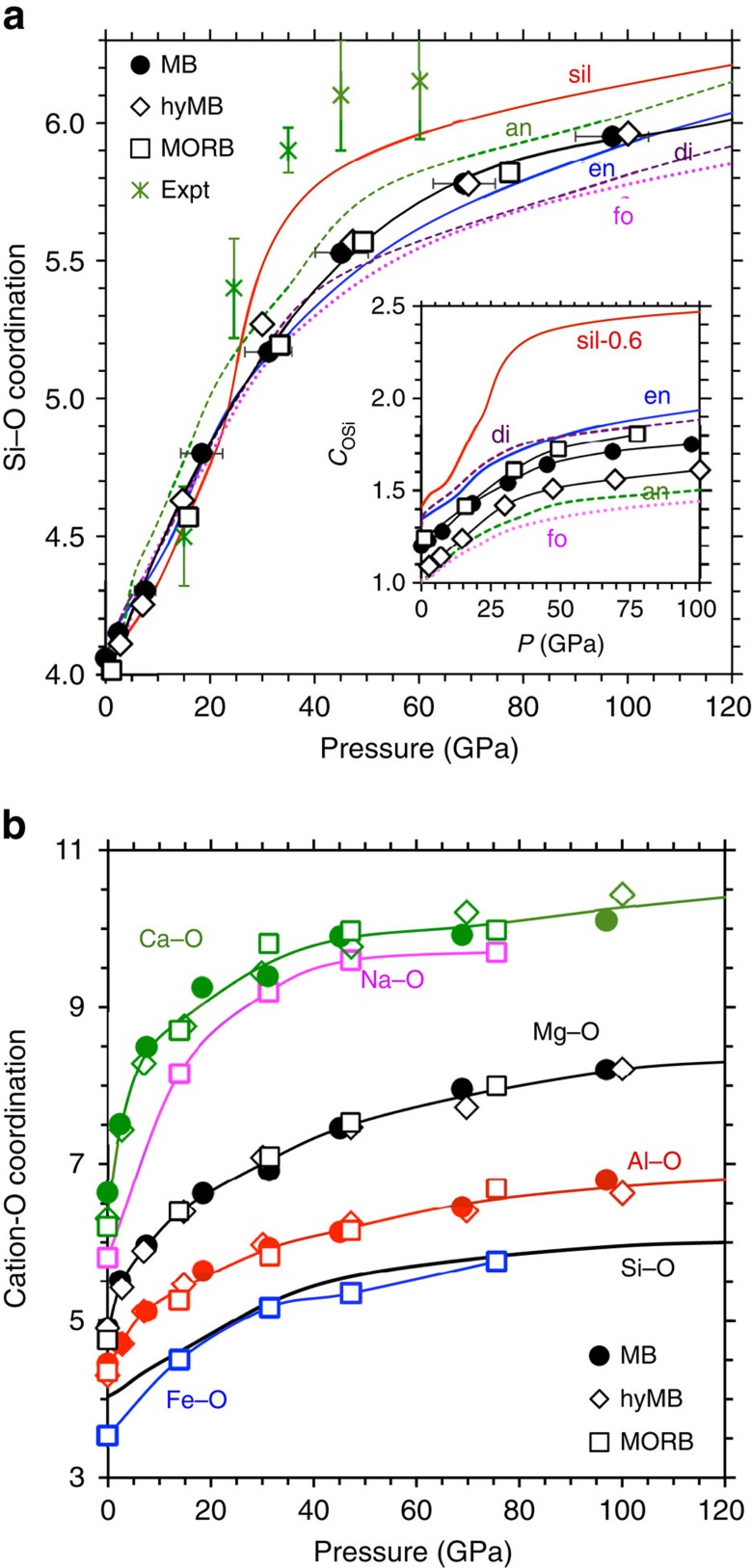
Mean cation–anion coordination. Their calculated values (symbols) are for MB, hydrous MB (hyMB) and MORB liquids. (**a**) Silicon–oxygen coordination (symbols and black line) compared with that for silica: sil (ref. 19), enstatite: en (ref. 12) , forsterite: fo (ref. 22), diopside: di (ref. 14) and anorthite: an (ref. 13). The experimental Si–O data (asterisks) are for molten basalt^9^. The inset shows the mean O–Si coordination for three basaltic liquids (symbols with black lines) and other liquids (the silica value shifted down by 0.6). (**b**) Coordination of Ca, Mg, Na, Fe and Al with respect to oxygen (symbols and lines). The coordination and pressure values are averaged over different temperatures at each volume for each liquid. Error bars in pressure represent the range covered by temperature 1800–4000 K on a given isochore.

The melt structure is largely controlled by cation–anion bonding so the structural changes due to pressure, temperature and composition can be better understood in terms of coordination environments consisting of different cations and anion. As pressure rises, the calculated mean Si–O coordination increases relatively rapidly initially from fourfold (at zero pressure) and then gradually at pressures above 30 GPa to sixfold (and eventually exceeding six) at high pressure in a remarkably similar way for all three basaltic compositions studied here (Fig. 1a). Other types of cation–anion coordination (Al–O, Ca–O, Fe–O, Mg–O and Na–O coordination) also increase with pressure with most changes occurring over narrower pressure intervals (Fig. 1b). For each case, the mean coordination remains almost unchanged on isochoric heating (Supplementary Fig. 1) though the distribution of coordination species widens (Supplementary Fig. 2). When the liquid is compressed, more high-coordination species appear at the expense of low-coordination species, and the overall coordination continuously increases on compression unlike abrupt coordination changes that occur in crystalline silicates. Though the calculated results are generally consistent with the experimental data^9^, the four- to sixfold Si–O coordination increases in the simulated liquids occur over wider pressure intervals than experimentally inferred. The calculated Fe–O coordination-pressure evolution is more gradual too, compared with the measured trend for molten fayalite^18^.

Comparisons with the previous first-principles simulations of several other liquids^12^,^13^,^14^,^19^ (Fig. 1a) suggest that local structural features are selectively dependent on composition. The mean Si–O coordination is, however, weakly sensitive to composition at all pressures with its values lying within 10% for different liquids including basaltic ones. This means that the corresponding structural units (coordination polyhedra) serve as building blocks of all silicate liquids with the abundances and stabilities of different coordination species depending on composition (Supplementary Fig. 2). The basalt melt contains >10% non-tetrahedral species at 0 GPa and 3,000 K, compared with nearly pure tetrahedral silica liquid^19^. On the other hand, the mean O–Si coordination is highly dependent on composition (inset of Fig. 1a), being sensitive to both water and Fe content. The water component systematically lowers the O–Si coordination at all conditions thereby depolymerizing the melt structure.

### Water speciation of hydrous melt

**Figure 2:**
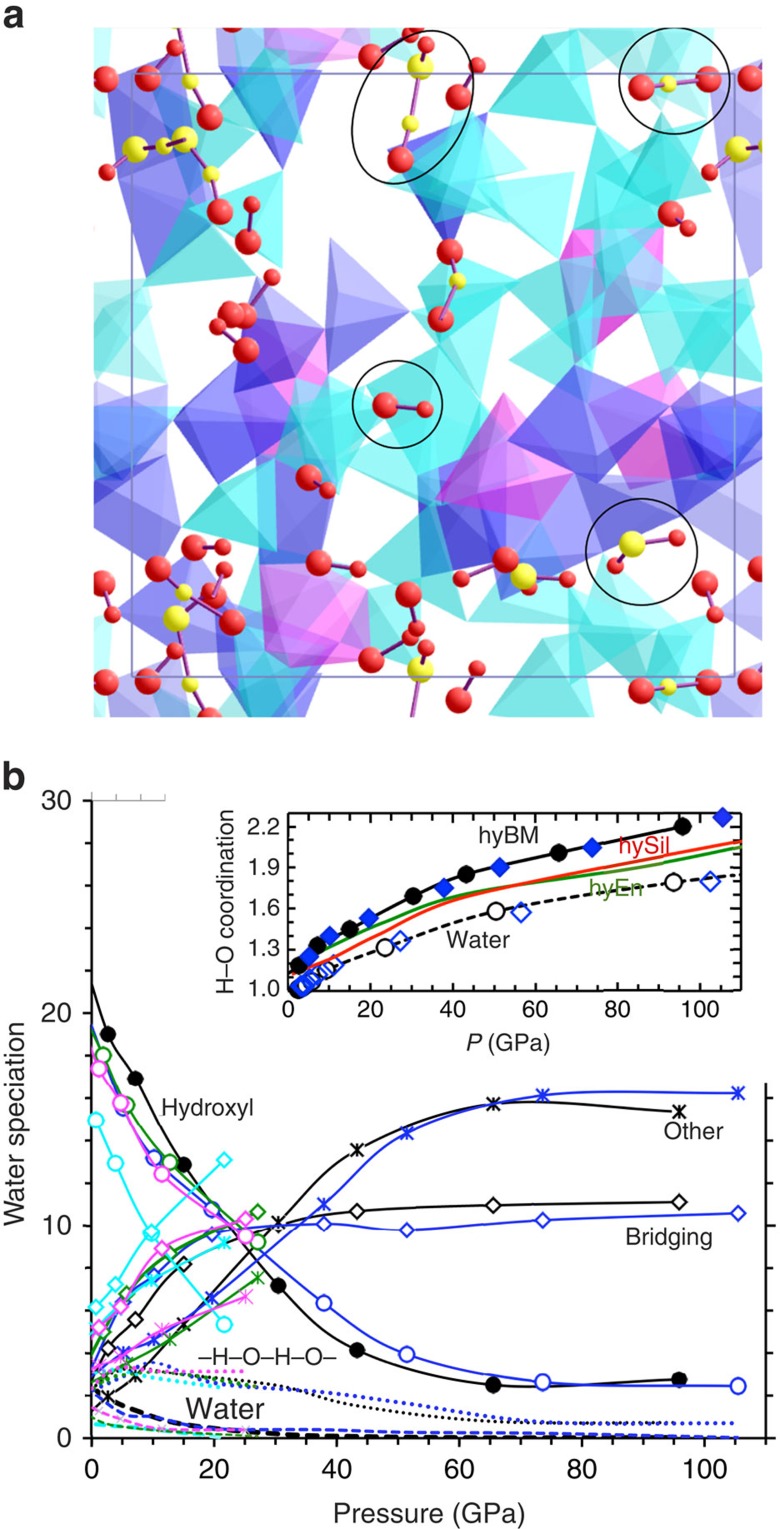
Speciation of water component in melt. (**a**) Visualization snapshot showing hydroxyls, free water molecule, polyhedral bridging and four-atom sequence (marked) in the melt at 3000 K and 7 GPa. The Si/Al–O coordination polyhedra and O–H bonding (large sphere–small sphere) are also displayed. (**b**) Abundances (expressed in terms of the number of H atom, 30 in total) of different forms of water speciation at 4,000 K (blue lines), 3000 K (black lines), 2,500 K (green lines), 2,200 K (magenta lines) and 1,800 K (cyan lines). Species grouped under ‘other' represent long chains. The inset shows the mean H–O coordination numbers of hydrous MB melt (hyMB) compared with that of pure water at 3,000 K (circles) and 4,000 K (diamonds) along with the results for hydrous silica (hySil) and enstatite (hyEn) melts.

How water (H_2_O) component dissolves in the melt impacts the host structure and properties. Our simulations of hydrous MB show that the speciation of water component occurring through oxygen–hydrogen bonding consists of various forms (Fig. 2a), whose proportions are sensitive to both pressure and temperature (Fig. 2b). Hydroxyls, water molecules and polyhedral bridging (–O–H–O–) together account for >90% of the speciation at zero pressure and 3,000 K with molecular water mostly bonded to Mg and Ca. This is consistent with the mean H–O coordination number of nearly one (Fig. 2b, inset). With increasing pressure, the abundances of polyhedral linkages with the appearance of polyhedral edge decoration and other extended forms (–O–H–O–H– chains, hydronium; Supplementary Fig. 3) increase as more oxygen gets bonded with hydrogen at the cost of isolated species. In compressed simulation supercell, available free volume may not be enough to accommodate polar molecular species anymore. The increased bonding activity is reflected in rapidly increasing mean H–O coordination, which exceeds 2 at pressures above 80 GPa. Interestingly, H–O coordination and H–O bond lengths for pure water are systematically lower than those for the hydrous melt (inset of Fig. 2b, Supplementary Fig. 4). Experimental evidences exist for some of the predicted speciation forms, in particular, hydroxyl, molecular water and edge decoration^20^,^21^.

### Thermal equation of state and density

The pressure–volume–temperature (*P–V–T*) results obtained from a series of liquid simulations (Supplementary Table 1) can be described by the Mie–Grüneisen form of equation of state: 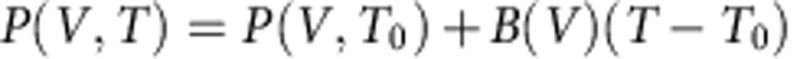. A fourth order Birch–Murnaghan equation is needed to accurately represent the reference isotherm at *T*_0_=3,000 K mainly because of initial high compressibility of the liquid, perhaps arising from large coordination changes occurring in the low-pressure regime. Based on the calculated coordination-pressure profiles (Fig. 1b), the oxygen coordination of Ca, Fe, Mg and Na (that is, network modifiers) apparently contributes to initial compression (up to 20 GPa) more than the Si/Al–O coordination does. Such coordination changes become gradual and all cation–anion bond distances start to systematically decrease on compression thereby making the liquid much stiffer at higher pressure. As shown in Table 1, the calculated equation-of-state parameters are in excellent agreement with those based on experiments for different melt compositions^9^,^11^. For each melt, increasing thermal pressure on compression is reflected by strongly volume dependent coefficient *B*. This behaviour can be further linked to the Grüneisen parameter, whose value increases nearly linearly from 0.2±0.1 to over 1.5±0.2 upon twofold compression with no discernable effects of Fe and water. This finding is generally consistent with the previous simulations^12^,^13^,^14^,^22^ and experimental inferences^23^,^24^,^25^.

**Figure 3:**
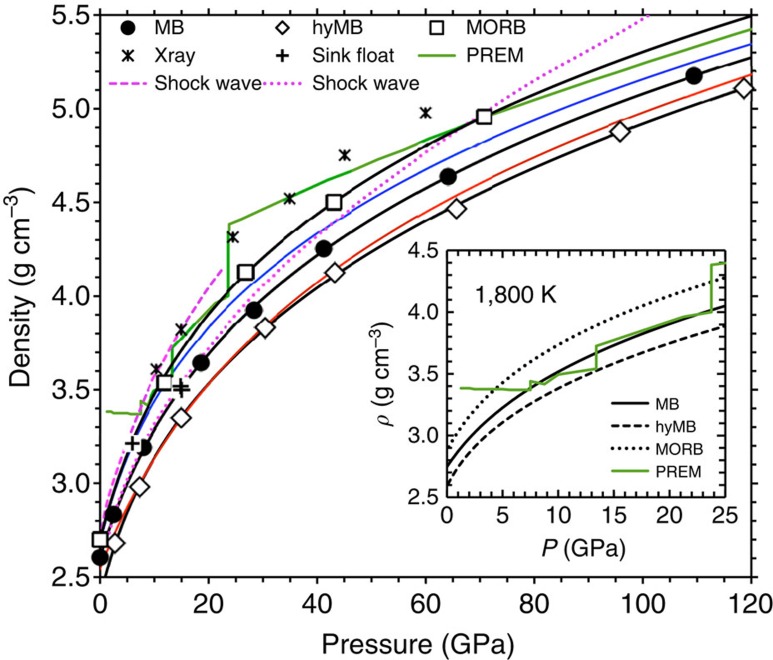
Melt density–pressure profiles. The calculated MB results are shown as the fourth order Birch–Murnaghan isotherms along 2,200 K (blue line), 3,000 K (black line with circles) and 4,000 K (red line). The results for hydrous MB (hyMB, black line with diamonds) and MORB (black line with squares) are along 3,000 K isotherms. The calculated densities are compared with the seismic data (PREM: Preliminary Reference Earth Model (ref. 32)). Various experimental data are shown for comparisons: X-ray diffraction data (asterisks) at 2200–3273 K (ref. 9), sink-float data (crosses) at 1,673 K and 5.9 GPa (ref. 26) and at 2,473–2,773 K and 15 GPa (ref. 27) for basalt/MORB composition, shock-wave isentrope measured for MB applied to basaltic composition (dashed lines) from ref. 7, and shock-wave 1,673 K isotherm for MB (dotted lines) from ref. 8. The inset compares the calculated density at 1,800 K of three melts with the mantle density (PREM) in the low-pressure regime.

The melt density–pressure isotherms predicted by the above Mie–Grüneisen equation diverge initially with increasing pressure, and then tend to remain parallel at high pressure (Fig. 3). Based on our simulation results, the MORB density is higher than the MB density, which is, in turn, higher than the hydrous basalt density at all pressure–temperature conditions. The Fe-induced increase in the melt density becomes bigger at higher pressure unlike relatively uniform density decrease caused by the water component. The near-MORB melt shows a higher densification rate than the other two melts studied here. The calculated melt densities compare favourably with the measured data from X-ray diffraction^9^, sink-float^26^,^27^ and shock-wave experiments^7^,^8^ (Fig. 3, Supplementary Fig. 5). It is interesting to note that the near-MORB density values along 3,000 K isotherm tend to roughly lie between the X-ray diffraction data at 2,200–3,273 K, and the sink-float data at 1,673 K and 2,473–2,773 K for basalt/MORB melts. Some systematic deviation shown by calculated results can be attributed partly to the compositional and temperature differences between the simulations and experiments.

### Melt-water solution properties

**Figure 4:**
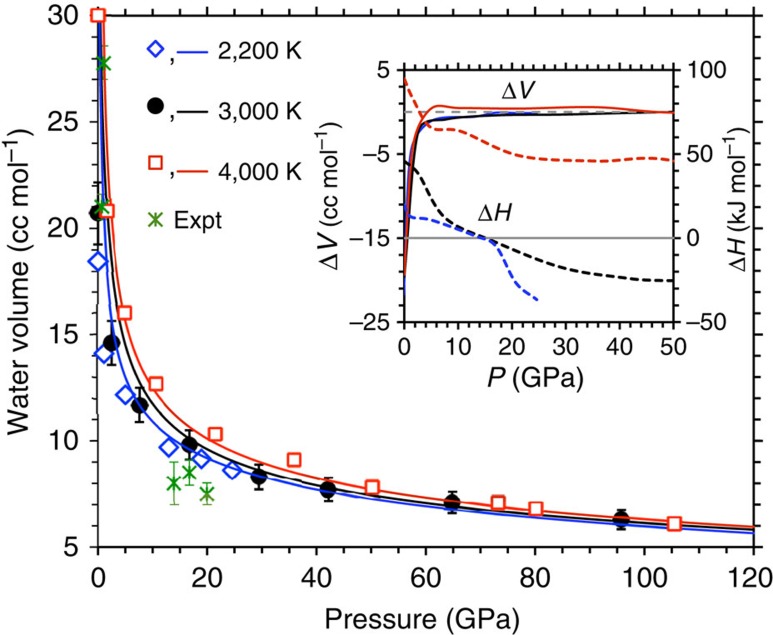
Volume and energetics of water-melt solution. The calculated partial molar volume of water in basaltic melt at 2,200 K (diamonds), 3,000 K (circles) and 4,000 K (squares) is plotted as a function of pressure compared with the experimental data^10^,^11^,^29^. The pressure–volume curves (lines) for the pure water are at the corresponding temperatures. The inset shows the estimated volume Δ*V* (solid lines) and enthalpy *ΔH* (dashed lines) of the simulated basalt melt-water solution as a function of pressure at three temperatures.

It is important to check whether the dissolved water behaves like other oxide components by having a well-defined partial molar volume in the melt. The apparent (partial) molar volume (
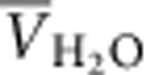
) of water in the basaltic melt obtained using the equation 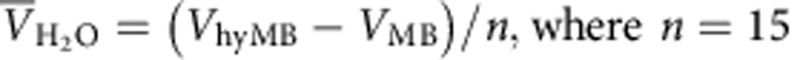, the number of water molecules present in the simulated hydrous MB (Fig. 4) is significantly smaller than the molar volume of pure water (
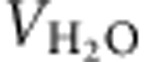
) at zero pressure. The two water volumes quickly approach each other as pressure increases. The volume of the melt-water solution defined as 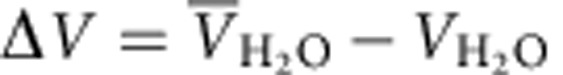 is thus large and negative at low pressure, the zero-pressure value being around −20 cm^3^ mol^−1^ over the temperature interval of 2,200 to 4,000 K. Its magnitude decreases rapidly initially with pressure to about zero above 10 GPa (Fig. 4, inset) so the melt-water solution becomes ideal within our computational uncertainty at high pressures along each isotherm. The predicted pressure-induced ideality can be rationalized as follows: The pure water perhaps is in the gaseous state (as characterized by over 95% singly oxygen coordinated H atoms and over 90% doubly hydrogen coordinated O atoms) and diffuses fast as individual water molecules at pressures below 5 GPa thereby covering relatively large distances (Supplementary Fig. 4). Available space is, however, highly squeezed within the melt so such molecular species cannot be easily accommodated. The compressed pure water is also structurally well connected and highly packed like the water is in the melt. This is reflected by increased coordination between H and O atoms consisting of over 30% twofold H–O and over 60% threefold O–H species at pressures above 25 GPa (inset of Fig. 2b).

**Figure 5:**
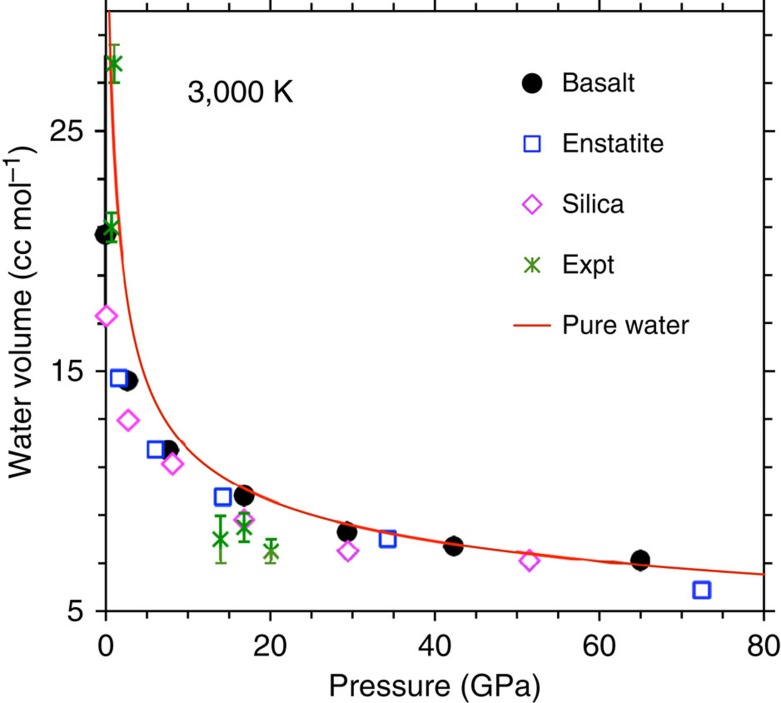
Different melt-water volumes. The calculated partial molar volumes of water in the hydrous basaltic liquid (this study) and previously studied hydrous enstatite^30^ and silica^31^ liquids are compared with the pure water volume (this study). Also shown are experimental data^10^,^11^,^29^ for the silicate melt-water.

The calculated 
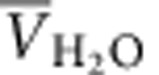
 values are comparable with the experimentally inferred values at low pressures^10^,^11^,^28^,^29^. Comparisons with the refined values from previous simulations of hydrous enstatite and silica liquids^30^,^31^ suggest that the solution properties are weakly dependent on the composition (Fig. 5). In particular, the partial molar volume of dissolved H_2_O for the hydrous basaltic liquid appears to be somewhat larger than that for other two liquids, with the hydrous silica liquid tending to be more non-ideal. These subtle differences arise primarily because the presence of the structure modifier cations in the basaltic melt facilitates the accommodation of water, particularly as hydroxyl groups and molecular water compared with that in the silica liquid.

The thermodynamic condition of the melt-water solution is set by the Gibbs free energy, 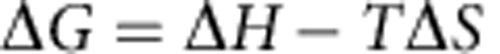, where the enthalpy of mixing (Δ*H*) can be obtained from the first-principles molecular dynamics simulations as 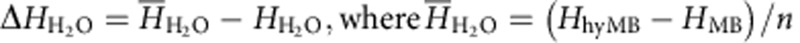 is the enthalpy per formula unit for the melt water, and 
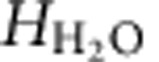
 is that for the pure water. As shown in the inset of Fig. 4, the calculated Δ*H* is positive and large at zero pressure for all temperatures so the hydrous silicate liquid readily devolatilizes at the ambient pressure. With increasing pressure, Δ*H* decreases by different extents at different temperatures, changing to negative above 10 GPa at 2,200 and 3,000 K. Such small and/or negative values of the enthalpy of solution over wide pressure range imply that the melt and water component are mostly miscible. To strictly confirm such miscibility requires that the entropy contributions be included, though a melt-water solution has higher entropy than the mechanical mixture.

## Discussion

The liquid–solid density crossover is possible in a multi-component silicate mantle mainly because of high compressibility and Fe enrichment of the liquid phase^10^,^11^,^28^. Our direct comparisons with seismically derived density profile^32^ indicate that the melt density can actually exceed the mantle density at one or more depths as shown in Fig. 3. The MORB density along the 3,000 K isotherm is higher than the mantle density around 14 and 23 GPa corresponding to the 410 and 670 km seismic discontinuities, and also at all pressures above 70 GPa. To explore this implication further, we estimated the melt density along the 1,800 K isotherm, which exceeds (for the MB and MORB) and approaches (for the hydrous melt) the mantle density at the 410 km depth. Our analysis further strengthens the hypothesis that dense melt could be buoyantly stable at those depths thereby providing a plausible explanation for low-velocity regions, consistent with several previous suggestions^4^,^10^,^11^,^26^.

Based on our calculations, the dissolved water being light component systematically lowers the melt density^10^,^11^,^28^,^33^. The calculated density contrast between the pure and hydrous melts is nearly independent of pressure and temperature. The basaltic melt density decrease per wt% water is 0.036 g cm^−3^, comparable to the estimated values of 0.035 and 0.030 g cm^−3^ for the enstatite and silica liquids^30^,^31^, respectively. It is thus remarkable that the water component can significantly influence the melt stability in the mantle irrespective of the composition. Based on our simulations, the densities of anhydrous and hydrous (with 5 wt% H_2_O) basaltic melts are 3.67 and 3.51 g cm^−3^, respectively, at the 410 km depth conditions of 13.4 GPa and 1,800 K, compared with the average mantle density^32^ of 3.54 g cm^−3^ at this depth (Fig. 3, inset). This means that a buoyantly stable melt layer at the base of Earth's upper mantle can be hydrous with a few (∼4) wt% dissolved H_2_O. The MORB density is larger than the anhydrous MB so more water can be accommodated in natural basalt melt so as to counter-balance the effects of Fe on the melt density. This is important because the presence of both the Fe and H_2_O components in substantial amounts usually facilitates mantle partial melting.

With constraints only at pure water, 5 wt% H_2_O content, and dry melt, it is not possible to establish ideal mixing of volume or miscibility. It may be that, by coincidence, a composition-dependent 
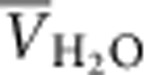
 matches the pure water value (
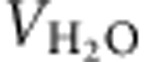
) when evaluated over the range from 5 wt% H_2_O to 0 studied here. This match is necessary, but not sufficient, to show constant partial molar volume of H_2_O across the full range of water content. So is the case of the predicted miscibility because arbitrarily high water content may not easily penetrate the silicate network, which has already been broken at lower water content like 5 wt% simulated here. Nevertheless, our analysis of melt-water system provides direct (first principles) evidence for the possibility that the water component shows ideal mixing of volume as well as high solubility (at least, up to 10 wt%, considering water contents of previously simulated hydrous enstatite and silica liquids^30^,^31^), in high-pressure silicate melts irrespective of the melt composition. Potential existence of water-rich melts over most of the mantle conditions in Earth's early history (a possible hydrous magma ocean) would have been a reservoir of water thereby making substantial contribution to the origin of hydrosphere^34^.

In summary, our first-principles molecular dynamics simulations of three basaltic melts (MB, hydrous MB and near-MORB compositions) represent a major step towards sampling natural magmas. The simulation results show that the effects of pressure, temperature and composition (Fe and water) on the melt structure and density are substantial. The simulated melt-water system behaves ideally with increased solubility at high pressure. Our analysis suggests that the silicate melts may be gravitationally stable in deep-mantle and potentially water-rich, perhaps serving as a water reservoir in Earth's early stages and presently as a hydrous melt layer at the 410 km depth.

## Methods

### Computational details

First-principles molecular dynamics simulations were performed within density functional theory using local (spin) density approximation (LDA) and projector augmented wave method using the Vienna ab initio simulation package (VASP)^35^. Previous studies have found that LDA works better than the generalized gradient approximation (GGA) for silicate and oxide materials^36^,^37^ as we have also assessed here the LDA/GGA differences on various melt properties (Supplementary Figs 1,4–6). Many simulations based on the canonical (*NVT*) ensemble were performed to explore compression from *V*/*V*_*X*_=1.5–0.5 covering the entire mantle pressure regime at 1,800–4,000 K, where *V*_*X*_=3422.5 Å^3^ is the reference volume. The numbers of atoms in the supercell were 244 (8 CaAl_2_Si_2_O_8_ and 14 CaMgSi_2_O_6_) for MB, 289 (with 5 wt% of water, that is, 15 H_2_O molecules) for hydrous MB and 234 atoms (with 9.9 wt% FeO and 2.4 wt% Na_2_O) for MORB (Supplementary Table 2).

For each composition, the initial structure was first melted at 6,000 K and then quenched down to 4,000 K and subsequently to lower temperatures at each volume. The simulations were performed for durations from 10 to 150 ps at different volume–temperature conditions with a time step of 1 femtosecond for MB and MORB, and 0.5 femtosecond for hydrous MB (Supplementary Fig. 7). The time averages of energy and pressure were computed by the blocking method^38^. We confirmed the liquid state of the simulated system by examining the mean-square displacement plots (Supplementary Fig. 8) and radial distribution functions (Supplementary Fig. 9). Fe-bearing basaltic liquid was simulated in low-spin (non-magnetic) state at five volumes using spin LDA (no Hubbard U term used), with the density differences with respect to the magnetic (high spin) state lying within 1% for the Fe content studied here. The effects of the Hubbard term on liquid density are anticipated to be very small based on our tests on (Mg,Fe)O (Supplementary Fig. 10). Finally, the pure water was simulated as a function of volume and temperature. The Pulay stresses arising from the use of a finite cutoff of 400 eV at Γ point were added as usual. Further details can be found elsewhere^12^,^13^,^14^,^19^,^22^. For the numbers of atoms used here, the finite system size effects on the calculated properties are expected to be negligible based on previous tests^36^.

### Structural analysis

The atomic coordination, which is often used to characterize the local structure, was calculated for a given species α with respect to another species β using


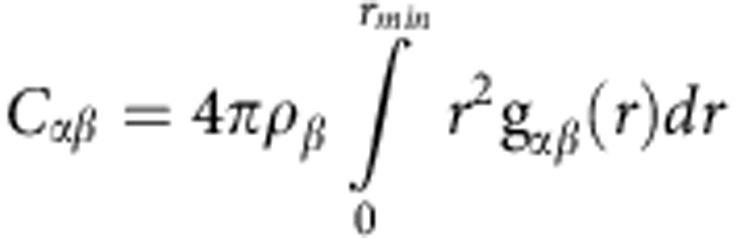


this nearest neighbour coordination is the number of contributing atoms (of species β), which lie within a spherical region centred at atom of species α and of radius defined by the corresponding *r*_min_ value (the minimum after the first peak in the respective radial distribution functions, see Supplementary Fig. 9). The simulated liquid phases show 25 (pure MB), 36 (hydrous MB) and 49 (MORB) types of coordination.

## Additional information

**How to cite this article:** Bajgain, S. *et al.* Structure and density of basaltic melts at mantle conditions from first-principles simulations. *Nat. Commun.* 6:8578 doi: 10.1038/ncomms9578 (2015).

## Supplementary Material

Supplementary Information Supplementary Figures 1-10, Supplementary Tables 1-2 and Supplementary References

## Figures and Tables

**Table 1:** Equation of state parameters.

	**MB**	**hyMB**	**Near MORB**	**Dry basalt (experiment)**	**Hydrous MORB (experiment)**
*ρ*_0_ (g cm^−3^)	2.61±0.02	2.38±0.02	2.70±0.03	2.48	2.46, 2.09
*K*_0_ (GPa)	27±2	18±1	23±1	24±2	13.9, 6.7
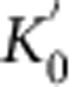	3.7±0.2	5.0±0.2	3.6±0.3	0.66±0.03	5.2, 5.7
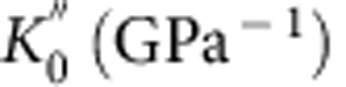	0.06±0.01	−0.19±0.05	0.12±0.04	−0.06±0.06	

The fourth order Birch-Murnaghan fit parameters for three basaltic melts (MB, hyMB and near-MORB) at 3,000 K, compared with their experiment-based values for dry basalt (MORB) melt^9^, and hydrous MORB melts with 2 and 8 wt% water^11^.

For all three melts studied here, *B*(*V*)=28.3(±0.3)–46.1(±1.0)*u*+18.6(±1.3)*u*^2^ (in the units of MPa/K), and γ(*V*)=3.2(±0.2)–3.0(±0.2)*u,* where *u*=*V*/*V*_0_=*ρ*_0_/*ρ.*
