# Supplementary material for: Structure and density of basaltic melts at mantle conditions from first-principles simulations
Source: Nat Commun. 2015 Oct 9;6:8578. doi: 10.1038/ncomms9578 (PMC4633951; doi:10.1038/ncomms9578)
Supplement: Supplementary Information — Supplementary Figures 1-10, Supplementary Tables 1-2 and Supplementary References [file ncomms9578-s1.pdf]

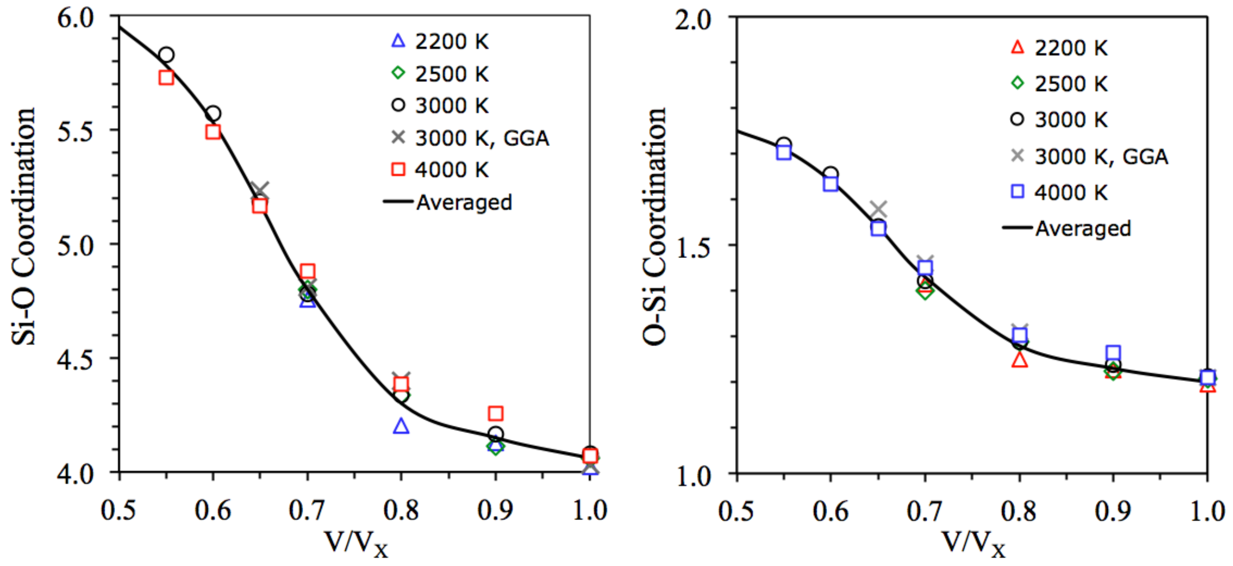

Supplementary figure 1: Mean coordination of Si and O atoms in the melts

Mean Si-O and O-Si coordination numbers as a function of compression (V/V_X, where V_X = 3422.5 Å^3) for the model basalt at 2200 K (triangles), 2500 K (diamonds), 3000 K (circles), and 4000 K (squares) with their average shown by the solid line. Also shown are the GGA results (crosses) for selected volumes at 3000 K, which are comparable with the main results obtained with LDA.

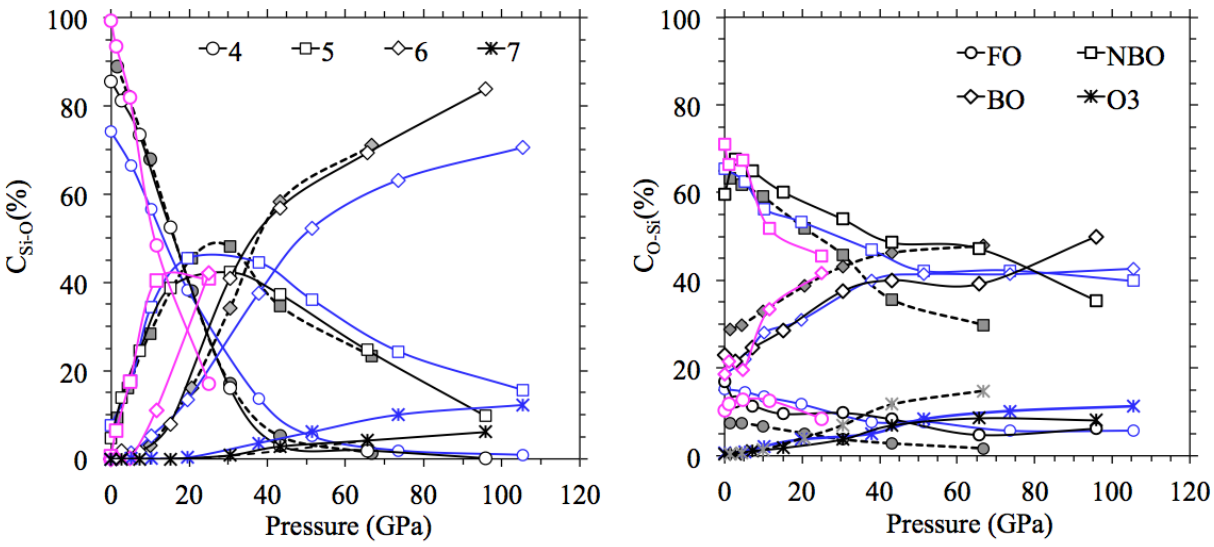

Supplementary figure 2: Coordination environment of Si and O atoms in melts

Abundances of various Si-O and O-Si coordination species as a function of pressure for the hydrous model basalt (hyMB) at 4000 K (blue lines), 3000 K (black lines), and 2200 K (magenta lines). In each case, symbols represent the calculated data points. The results for the pure model basalt (MB) are shown only at 3000 K (dashed lines with grey symbols). While the Si-O coordination distribution is similar between dry and hydrous melts, the O-Si coordination distribution differs between the two melts. The water content decreases the number of bridging oxygen (BO) and tends to increase the proportions of free oxygen (FO) and non-bridging oxygen (NBO). Also, the oxygen tri-clusters (O3) become more abundant at higher *P*.

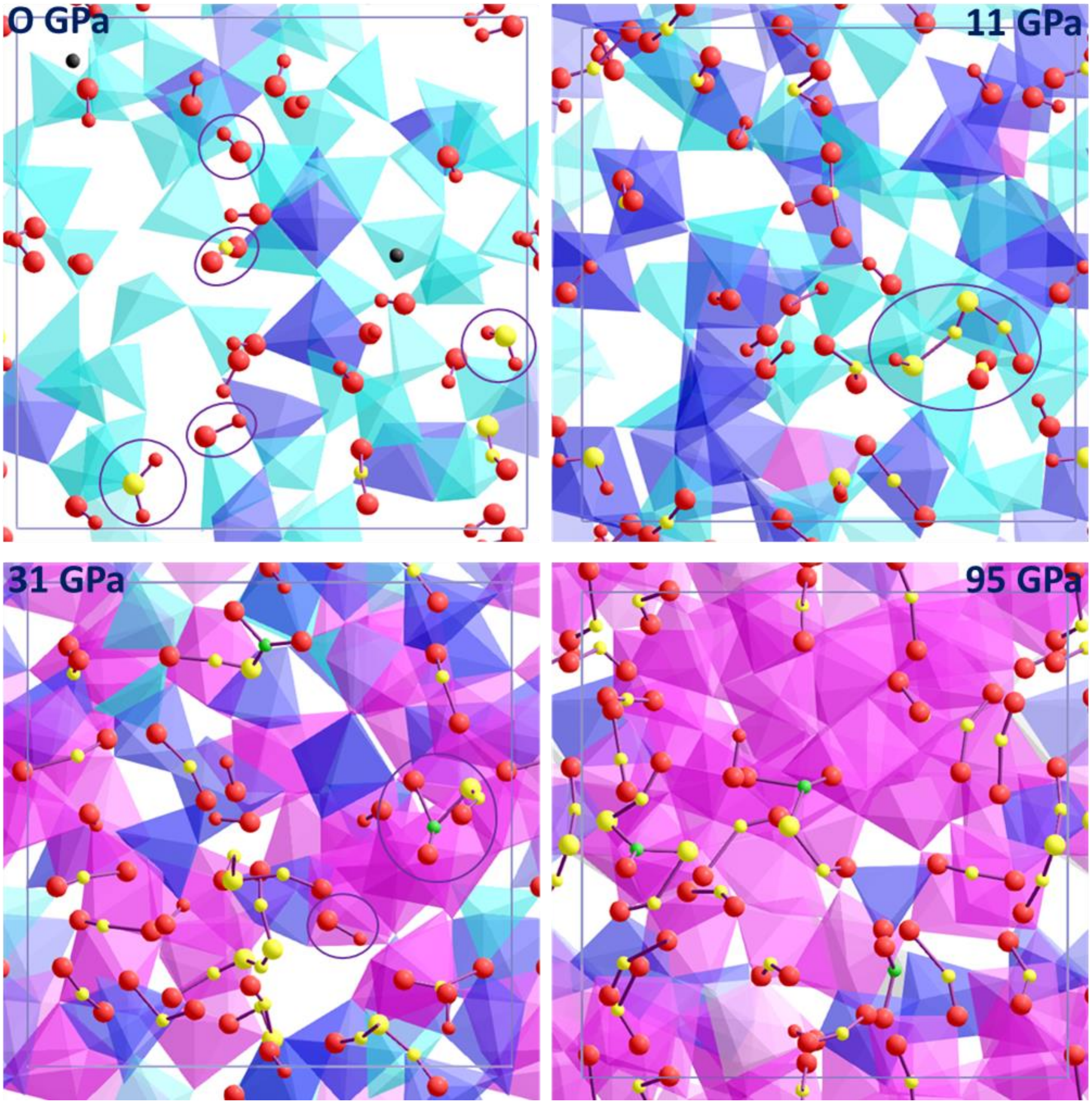

Supplementary figure 3: Water speciation in melt under different pressures

Visualization snapshots of water (H_2O) speciation of hydrous model basalt melt at 3,000 K and different pressures generated by the atomistic visualization system[1]. The cyan, blue, purple, and white polyhedra represent four-, five-, six-, and seven-fold Si/Al-O coordination species, respectively. The H atoms (small spheres) and O atoms (large spheres) are colored yellow, red, and green, respectively, if they are bonded to one, two, and three O atoms or H atoms. Ellipses/circles highlight common species such as O-H, O-H-O, and H_2O.

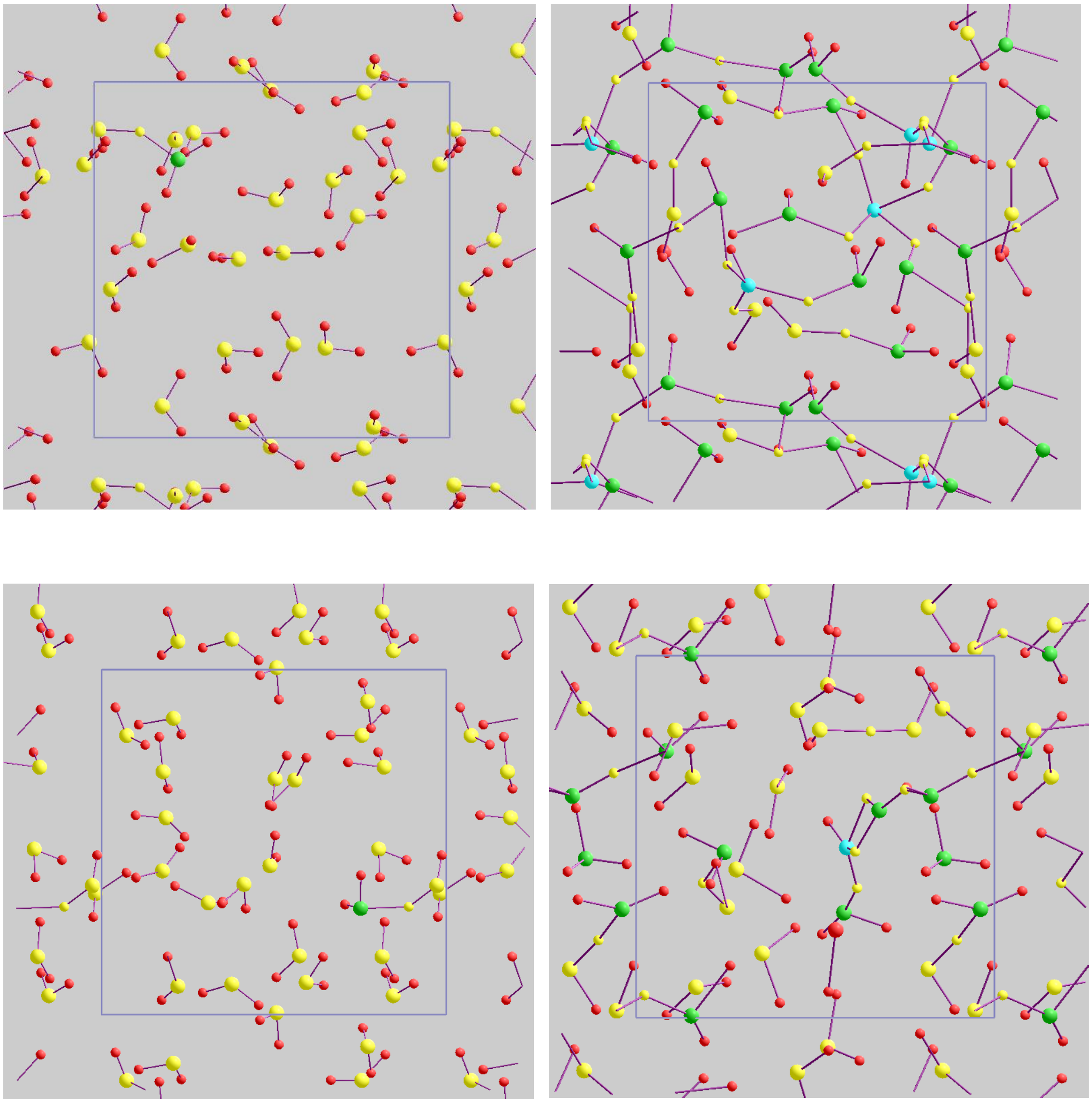

Supplementary figure 4: Visualization snapshots of simulated pure water

The structures of pure water at 3000 K at zero pressure (left) and 30 GPa (right) predicted by LDA (upper two images) are comparable with the GGA prediction (lower two images). Small and large atoms represent H and O atoms with O-H bonds shown. The color of the spheres encode the coordination state of the corresponding atom with respect to the atoms of other species: black = 0, red = 1, yellow = 2, green = 3, and cyan = 4. At the zero pressure, the simulated water consists of nearly 100 percent water molecules (small red-large yellow- small red triplets). At high pressure, O atoms are bonded with three or more H atoms, and also H atoms are bonded with two or more O atoms. The LDA structure at high pressure contains more high-coordination species compared to the GGA one.

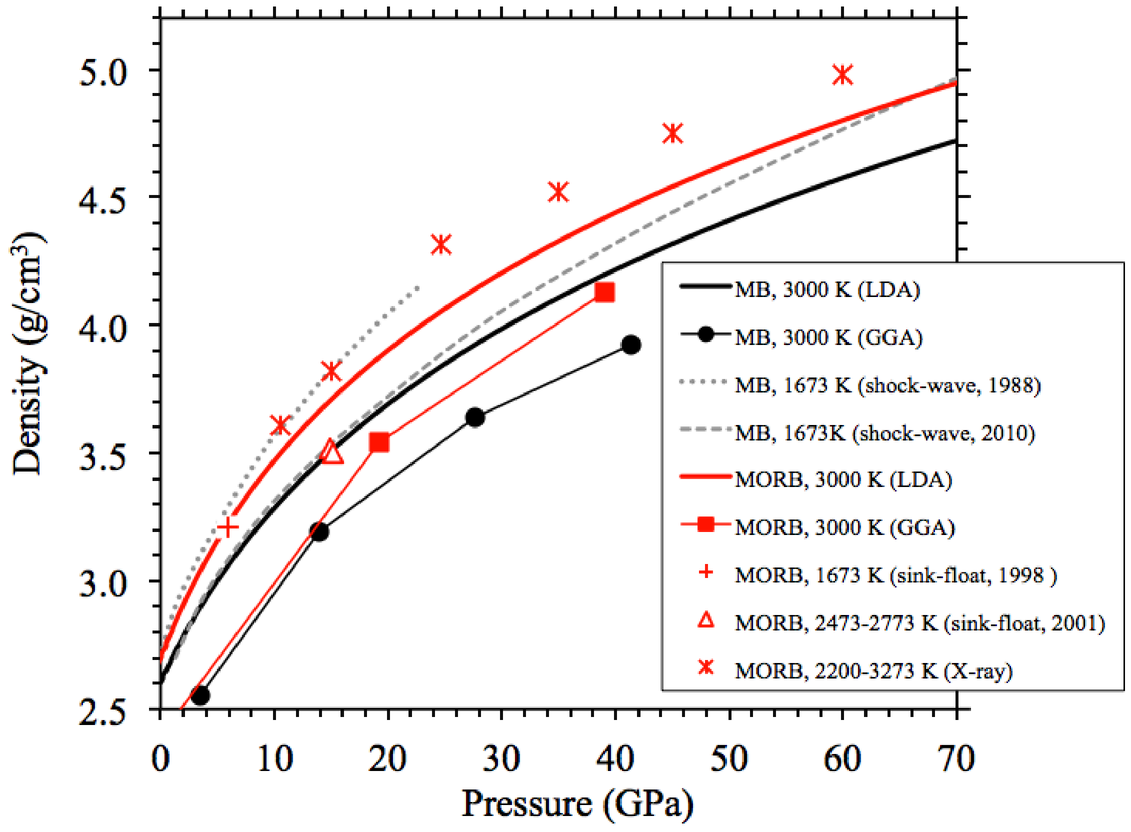

Supplementary figure 5: Calculated melt densities compared with the experimental data

The calculated melt densities for model basalt (MB) and MORB along 3000 K isotherms (solid thick lines) obtained using local density approximation (LDA) are compared with those (symbols with thin lines) obtained using generalized gradient approximation (GGA). The GGA densities are systematically lower than the LDA densities by a few percent. While the measured data from X-ray diffraction[2], sink-float[3,4], and shock-wave[5,6] experiments are not fully consistent with each other, the LDA results compare better with the experimental data for both model basalt and MORB liquids than the GGA results.

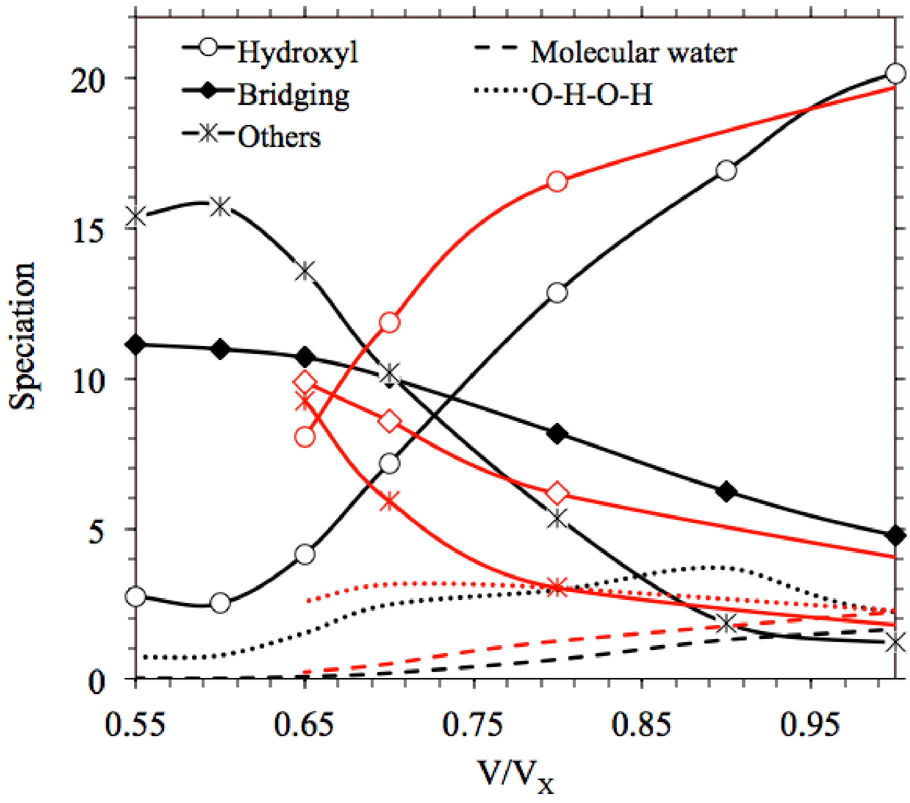

Supplementary figure 6: Water speciation predicted by LDA versus GGA

Abundances (expressed in terms of the number of H atom, 30 in total) of different forms of water speciation predicted with LDA (black lines) and GGA (red lines) at 3000 K as a function of compression (V/V_X, where V_X = 3422.5 Å^3). Species grouped under "others" represent long chains. The LDA and GGA results show equally rich set of species and comparable trends with compression.

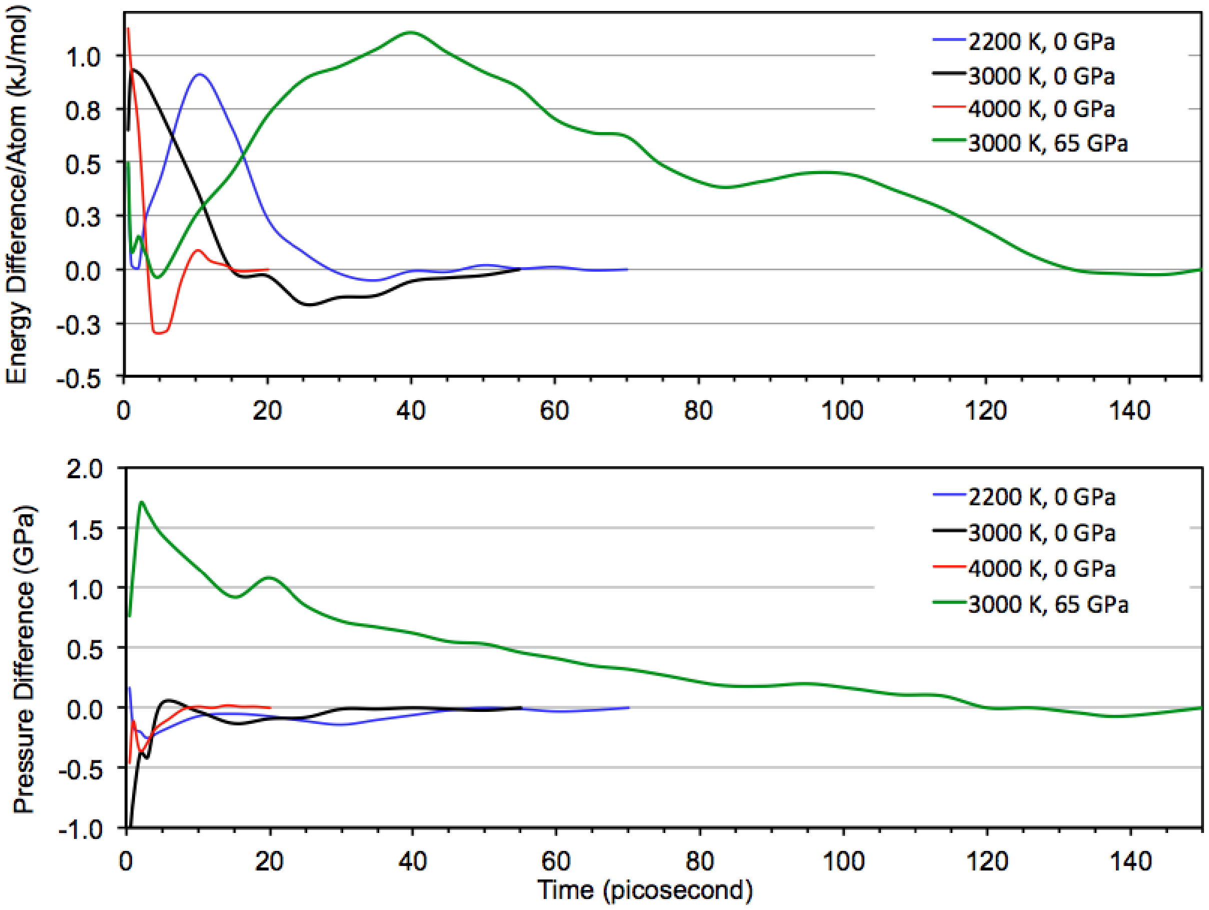

Supplementary figure 7: Time-convergence analysis of calculated energy and pressure

Time evolution of the energy and pressure differences for model basalt melt from the values obtained by averaging over the full simulation durations at different conditions shown. The results converge well relatively fast at zero pressure for all temperatures. However, the convergence is rather slow at high pressure. At 3000 K and 65 GPa, the energy obtained for 100 ps differs from the value obtained for longer run of 150 ps by less than 0.5 kJ/mol or 5 meV per atom. Similarly, the pressure converges within 0.3 GPa.

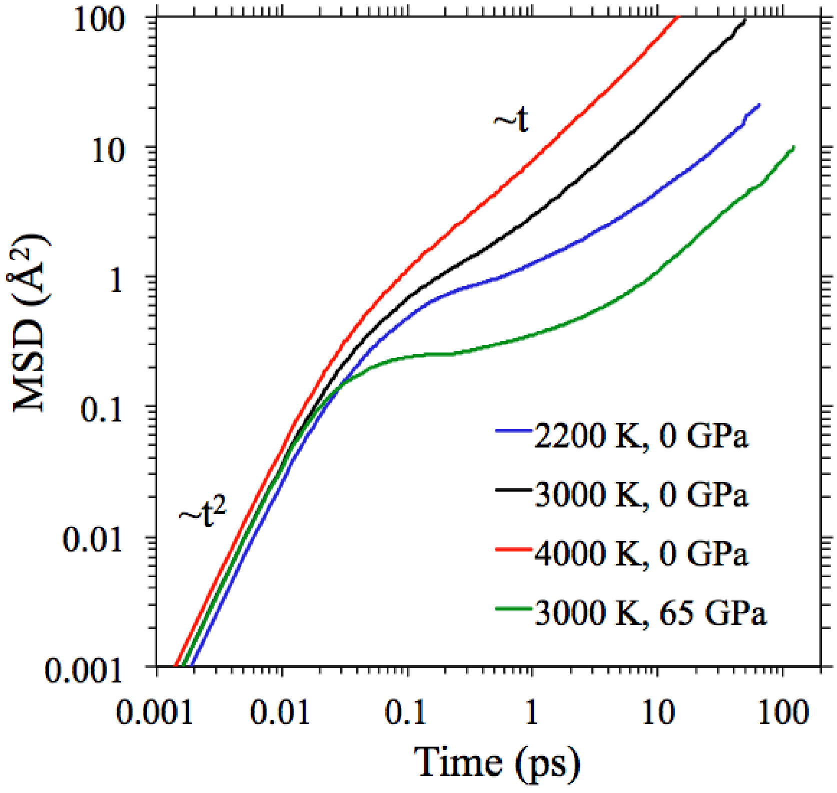

Supplementary figure 8: Mean squared displacement plots at different conditions

Mean square displacement (MSD) as a function of time for all species considered together (total MSD) for model basalt melt at four conditions as shown. In diffusive regime, MSD-time shows a slope of unity in the log-log plot.

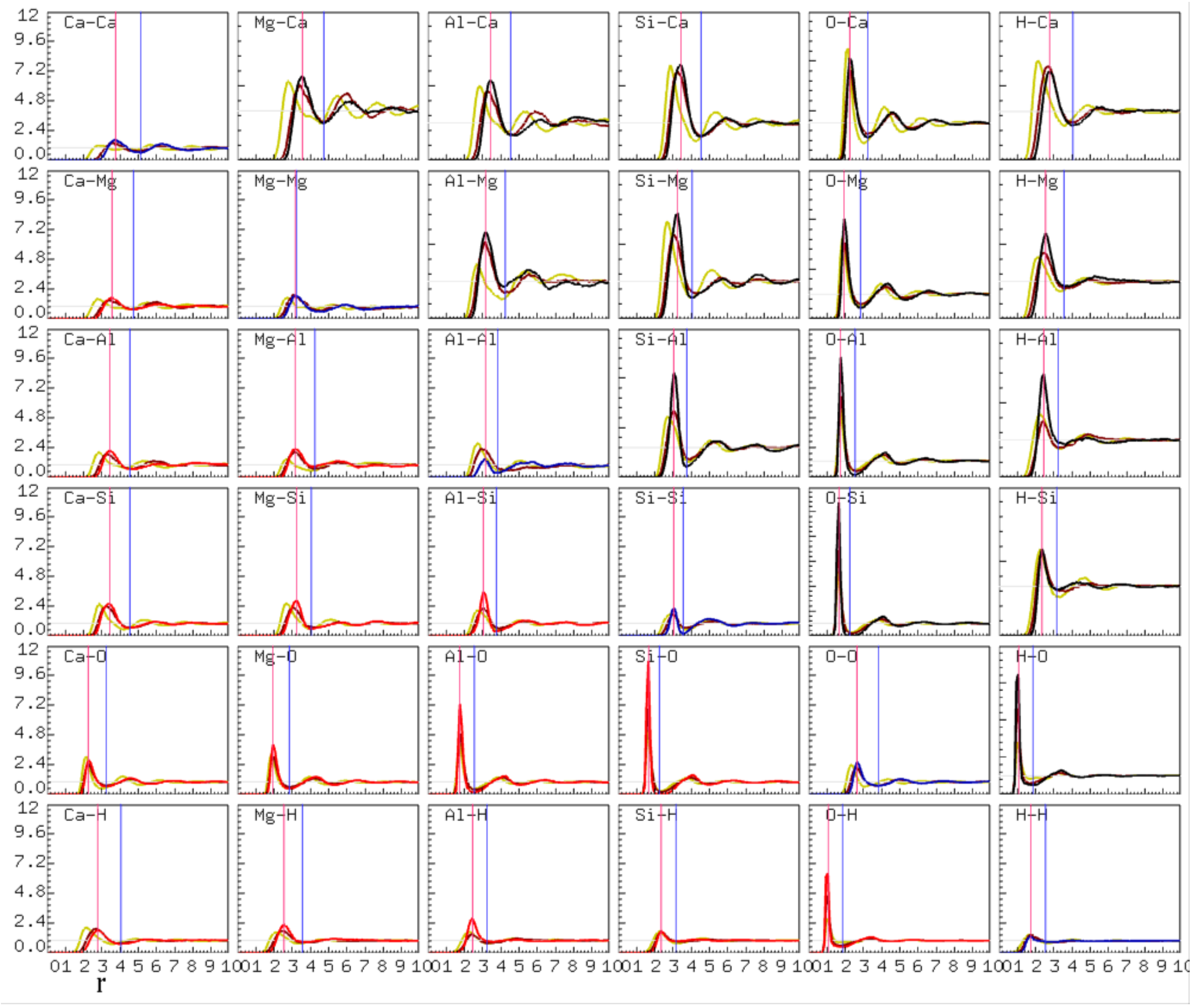

Supplementary figure 9: Full set of partial radial distribution functions

RDF matrix (symmetric) plot at three different conditions for hydrous model basalt melt: 0 GPa, 1800 K (black lines), 10 GPa, 3000 K (brown lines) and 105 GPa, 4000 K (yellow lines). The vertical lines mark the first peak (red line) and the minimum after the first peak (blue line).

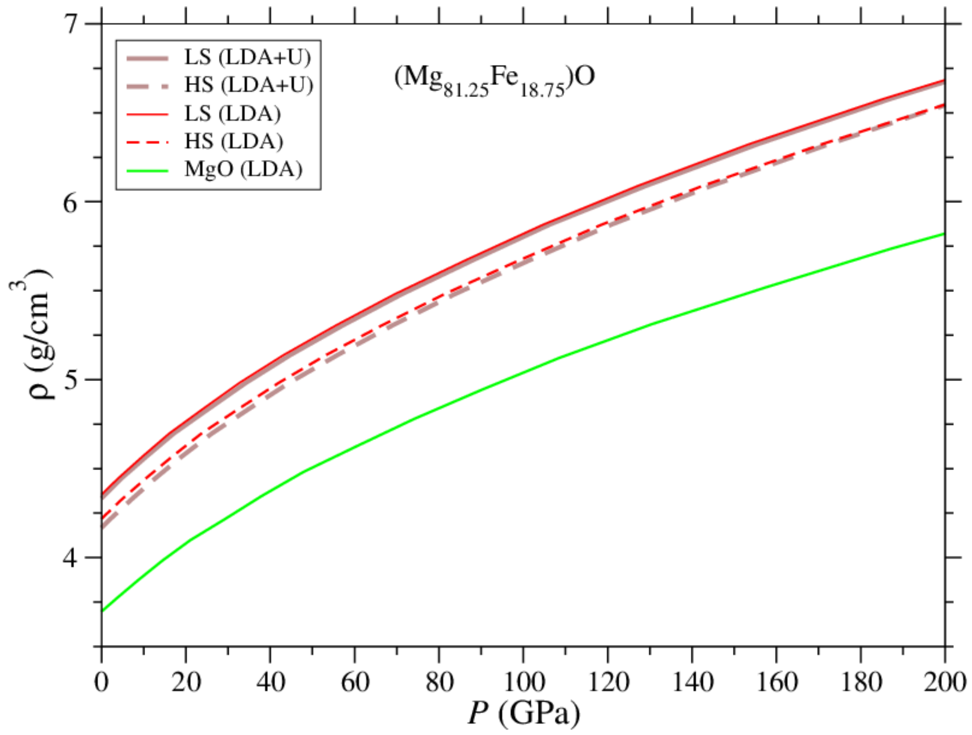

Supplementary figure 10: Comparison between LDA and LDA+U calculations

Density-pressure profiles for pure MgO, and (Mg,Fe)O with 18.75 wt% Fe at 0 K using spin local density approximation (LDA). Low spin (LS) and high spin (HS) results were obtained using LDA and LDA+U (with U = 5.5 eV). Note that the effects of using Hubbard (U) term on density are almost negligible.

Supplementary Table 1

The calculated pressure (*P*) and total energy (*E*) at different volumes for three temperatures for (dry) model basalt used for equation of state fit are given. The uncertainties lie within 0.3 GPa for pressure and 5 meV/atom for energy.

	2200 K		3000 K		4000 K	
V (Å^3)	*P* (GPa)	*E* (eV)	*P* (GPa)	*E* (eV)	*P* (GPa)	*E* (eV)
3422.5	-0.93	-1881.9	-0.49	-1837.5	1.15	-1779.1
3080.2	1.2	-1887.7	2.4	-1843.4	4.5	1787.3
2738.0	5.3	-1887.8	7.9	-1845.0	10.7	-1792.2
2396.0	14.6	-1883.8	18.6	-1843.3	23.3	-1790.9
2300.0	17.7	-1880.1				
2224.6	22.4	-1872.6	28.4	-1834	33.8	-1782.7
2053.5			41.2	-1821.4	49.1	-1769.4
1882.4			64.1	-1787.5	72.7	-1728.1
1687.4			109.5	-1708.6	117.2	-1660.5

Supplementary Table 2

Compositions of three basaltic (model basalt, hydrous model basalt, and near MORB) melts simulated in this study compared to experimentally studied (real) molten basalts in terms of wt% of oxide components. Model basalt represents eutectic composition of 36 wt% anorthite and 64 wt% diopside. MORB represents mid-ocean ridge basalt composition. We considered 5 wt% water on the basis of experimentally studied hydrous melts: 2 and 8 wt% in hydrous MORB[7] and 5 wt% in hydrous melt[8].

	Model Basalt (MB)	Hydrous Model Basalt (hyMB)	Near MORB	Basalt	Dry MORB
CaO	23.5	22.3	12.1	12.4	11.7
MgO	10.73	10.2	7.9	7.9	7.9
Al_2O_3	15.5	14.8	16.0	15.2	16.0
SiO_2	50.3	47.8	51.8	50.2	51.8
FeO			9.9	7.8	10.0
Na_2O			2.4	2.3	2.7
H_2O		4.9			
Others				3.5	3.3
	100	100	100	99.8	99.8
Total Number of Atoms	244	289	234		

Supplementary references

1. Bhattarai, D. Karki, B. B. Atomistic visualization: Space-time multiresolution integration of data analysis and rendering. *J. Mol. Graph. Model.* 27, 951-968 (2009).

2. Sanloup, C., Drewitt, J. W. E., Crepisson, C., Kono, Y., Park, C., McCammon, C., Hennet, L., Brassamin, S., Bytchkov, A. Structure and density of molten fayalite at high pressure. *Geochim. Cosmochim. Acta* 118, 118-128 (2013).

3. Agee, C. B. Crystal-liquid density inversions in terrestrial and lunar magmas. *Phys. Earth Planet. Int.* 107, 63-74 (1998).

4. Ohtani, E., Maeda, M. Density of basaltic melt at high pressure and stability of the melt at the base of the lower mantle. *Earth Planet. Sci. Lett.* 193(1), 69-75 (2001).

5. Rigden, S. M., Ahrens, T. J., Stolper, E. M. Shock compression of molten silicate: Results for a model basaltic composition. *J. Geophys. Res.: Solid Earth* 93(B1), 367-382 (1988).

6. Asimow, P. D., Ahrens, T. J. Shock compression of liquid silicates to 125 GPa: The anorthite- diopside join. *J. Geophys. Res.: Solid Earth* 115(B10) (2010).

7. Sakamaki, T., Suzuki, A., Ohtani, E. Stability of hydrous melt at the base of the Earth's upper mantle. *Nature* 439, 192-194 (2006).

8. Matsukage, K. N., Jing, Z., Karato, S. Density of hydrous silicate melt at the conditions of Earth's deep upper mantle. *Nature* 438(7067), 488-491 (2005).
